# A pilot effectiveness study of the Enhancing Parenting Skills (EPaS) 2014 programme for parents of children with behaviour problems: study protocol for a randomised controlled trial

**DOI:** 10.1186/s13063-015-0741-y

**Published:** 2015-05-20

**Authors:** Margiad Elen Williams, Judy Hutchings

**Affiliations:** Centre for Evidence Based Early Intervention, Nantlle Building, Normal Site, Bangor University, Bangor, Gwynedd LL57 2PZ UK

**Keywords:** Behaviour problems, Randomised controlled trial, Parent training, Health visitors

## Abstract

**Background:**

The Enhancing Parenting Skills (EPaS) 2014 programme is a home-based, health visitor-delivered parenting support programme for parents of children with identified behaviour problems. This trial aims to evaluate the effectiveness of the EPaS 2014 programme compared to a waiting-list treatment as usual control group.

**Methods/Design:**

This is a pragmatic, multicentre randomised controlled trial. Sixty health visitors will each be asked to identify two families that have a child scoring above the clinical cut-off for behaviour problems using the Eyberg Child Behaviour Inventory (ECBI). Families recruited to the trial will be randomised in a 1:1 ratio into an intervention or waiting-list control group. Randomisation will occur within health visitor to ensure that each health visitor has one intervention family and one control family. The primary outcome is change in child behaviour problems as measured by the parent-reported ECBI. Secondary outcomes include other measures of child behaviour, parent behaviour, and parental depression as measured by parent-reports and an independent observation of parent and child behaviour. Follow-up measures will be collected 6-months after the collection of baseline measures.

**Discussion:**

This is the first rigorous evaluation of the EPaS 2014 programme. The trial will provide important information on the effectiveness of a one-to-one home-based intervention, delivered by health visitors, for pre-school children with behaviour problems. It will also examine potential mediating (improved parent behaviour and/or improved parental depression) and moderating (single parent, teenage parent, poverty, low education level) factors.

**Trial registration:**

Current Controlled Trials ISRCTN06867279 (18 June 2014)

## Background

Behaviour problems in young children are increasing. In the UK, one child in five is affected by emotional and behavioural problems [1]. Studies have identified a number of risk factors associated with these problems, including poor parenting, poverty, and living in a single-parent household [1]. Children from disadvantaged backgrounds are more likely to start school lacking essential capabilities such as emotional regulation and social skills and with lower cognitive abilities [1]. Longitudinal studies have found that behaviour problems in early childhood are a precursor for adverse outcomes in adolescence and adulthood, including criminality, unemployment, substance misuse, mental health problems and teenage pregnancy [2]. Disadvantaged families are those most in need of intervention; however, they can be ‘hard to reach’ because of their lack of engagement with services and/or difficulties in accessing services.

Early intervention, in the pre-school and early school years, is an important way of tackling child behaviour problems before they become entrenched and whilst parents still have significant influence over children. Poor parenting is one, possibly the most significant, of the risk factors for child behaviour problems [3]. There is clear evidence that group-based parenting programmes are effective in both the treatment and prevention of child behaviour problems [4] and in helping parents to support children with a variety of developmental challenges including attention-deficit hyperactivity disorder (ADHD) [5, 6]. However, there are many families of young children for whom group-based programmes are either inappropriate or inaccessible. Disadvantaged parents are more likely to have low self-esteem and can sometimes find group environments daunting and fear being blamed for the child’s problems and criticised for poor parenting. Alternative modes of programme delivery including one-to-one, home-based interventions may be more suitable for disadvantaged families and a meta-analysis has shown that for these families individually-delivered programmes were superior to group-based programmes in terms of both parent and child outcomes [7].

### The role of a health visitor

In the UK, health visitors are public health nurses working with young children and families. They are the only health professionals that have universal access to, and responsibility for, all children from birth to 4 years old. They play a key role in promoting child health and development through the Child Health Promotion Programme (CHPP), the core health service for promoting, protecting, and improving the health and well-being of children and families [8]. Their main priorities are to prevent social exclusion in children and families, to tackle key public health issues such as obesity and smoking, to promote infant, child, and family mental health, and to support the capacity for better parenting [9]. There are two components to their work with families: universal and targeted. The universal service for all families is delivered through the CHPP and includes support throughout pregnancy and the first year of life, and monitoring the development and health of children to age 4. The targeted component of their work supports vulnerable families through intensive home- visiting that includes parenting interventions for parents of young children who are displaying behavioural difficulties. However, there is currently no standard model of intervention for child behaviour problems, so the services that parents are receiving can vary widely depending on their level of needs [9].

### Evidence for home-based/home-visiting parenting programmes

A home-visiting programme is a service delivered to vulnerable families within their own homes. It can include emotional support, providing access to other services, and direct instruction on positive parenting skills. Home-visiting programmes vary widely in content, the range of services offered, the age of the target child, and frequency/intensity of home visits. Several reviews have been published and the majority conclude that home-visiting programmes benefit families on a variety of outcomes, including improvements in parental behaviour [10–13], child abuse and neglect [10, 12], child behaviour/temperament [10].

Other reviews have looked at the components of home-visiting programmes to establish which are most important for successful outcomes [14, 15]. Components of successful programmes include a strengths-based approach, a structured curriculum, and experienced home visitors. Previous research has shown that when a home-visiting programme is delivered by experienced nurses, as opposed to paraprofessionals, benefits to families are sustained in the long-term [16].

Previous research has shown that health visitors are ideally placed to support ‘hard to reach’ families [10]. They have many of the necessary skills to work with these families and can detect problems such as poor parenting at an early stage [17]. There is some evidence that health visitors can effectively deliver home-visiting programmes [10, 17] and also parenting programmes for parents of children with behaviour problems [17–19]; however, most of the evidence comes from group-based parenting programmes, which may exclude many ‘hard to reach’ families due to access difficulties, lack of crèche/child care, and stigmatisation [20, 21].

### Development of the Enhancing Parenting Skills programme

The Enhancing Parenting Skills (EPaS) programme is based on work in the 1990s to develop a treatment programme for families of children with severe behavioural problems. The premise of the programme is that each child and family situation is unique and individualised support is the cornerstone of effective work with families experiencing such difficulties with their child. The EPaS programme supports parents as change agents. Parents of young children are often with them for much of the time and the child’s everyday environment has the biggest effect in terms of both contributing to problems and in helping children to learn adaptive behaviours. The main goal is to engage families in shared problem-solving aimed at empowering them to meet achievable goals. To do this professionals need skills in engaging and retaining families and the knowledge upon which to identify effective intervention strategies. This is why health visitors are ideal for this role.

The EPaS programme includes a standardised assessment procedure and structured case analysis formulation process to facilitate the identification of problem behaviours, their functions, and the necessary replacement behaviours. It incorporates elements of Goldiamond’s constructional approach that views behaviours as functional, or serving a purpose, in that they successfully produce a desirable or rewarding consequence [22, 23]. It also emphasises the importance of identifying the family’s assets and skills that can support the desired changes and goals for intervention. The interventions themselves are not standardised, although they are based on behavioural principles and work undertaken with families is extremely varied. The behavioural principles cover well-established child management skills such as strategies to enhance relationships through attending to the child, increasing desired behaviour through positive reinforcement, providing clear instructions, use of planned ignoring and limit setting. These components are the core of many effective parenting programmes [24–26]. A recent meta-analysis has shown that programmes that include attending to a child and positive reinforcement of desired behaviour are associated with larger effect sizes for both parent and child outcomes [27]. When parents are taught these skills through the use of role-play, this is also associated with large effect sizes. Importantly, due to the individualised delivery of the EPaS programme, parents are able to practice skills with their own children, which is also related to better outcomes [27]. Other research has looked at the social validity of parent training and shown that parents rate components such as attending, rewarding, ignoring, and instruction giving as acceptable and useful [28, 29].

### Evidence for the Enhancing Parenting Skills programme

The first evaluation of the EPaS programme included parents of children with severe behavioural problems who were referred to a Child and Adolescent Mental Health (CAMHS) service. This intensive treatment programme produced excellent long-term (4-year) results relative to the standard CAMHS treatment group, in terms of statistically significant improvements in child behaviour, reduced maternal depression and increased use of positive parenting skills [30, 31]. Results also demonstrated significantly lower service use 4 years postintervention for the intensive treatment (IT) group [32].

Although the intensive EPaS programme was effective, it was only accessible to those parents who had a child referred to CAMHS and, in the late 1990s, at the time of the development of CAMHS primary care services the core content of the programme was re-developed for health visitors [33]. In a small trial with 24 health visitors and 36 families each health visitor identified 1 family to work with and attended weekly half-day workshops for 10 weeks. Twelve health visitors also identified a control family presenting with similar problems. Results showed significant improvements in child behaviour and parental mental health only for the intervention group with non-significant changes for the control families. Health visitors demonstrated increased knowledge of behavioural principles and increased use of behavioural intervention skills, including observation strategies, keeping detailed records, case analysis and encouraging families to participate in record keeping. Health visitors also reported satisfaction with the course [33].

These results for the EPaS programme were promising; however, the training programme was intensive and time-consuming for health visitors and, therefore, not particularly practical for real-world implementation. In 2012, the training was revised into a 2-day course with additional material developed to support the programme in a trial funded by the Waterloo Foundation (WF). The aim of this trial was to train staff across Wales in evidence-based principles for one-to-one work with common behavioural difficulties in children with developmental difficulties (for example, sleeping, eating, and toileting difficulties as well as tantrums). Results from the WF trial were again promising, with significant reductions in child behaviour problems, negative parenting styles, and significant improvements in parental well-being [34]. However, attendee feedback suggested that 2 days was not sufficient time to cover the course content thoroughly and, furthermore, because the course was delivered to a wide range of intervention staff, many attendees did not have the necessary child development knowledge or access to families.

The result of the WF trial was to further revise the programme to address the limitations of previous trials. This involved a decision to, once again, target the programme on health visitors, to extend the training from 2 to 3 days, and to further expand the manual to include more detail regarding the 3 phases of the programme: assessment, case analysis and intervention. Health visitors are ideally suited to deliver the programme since they have the necessary child developmental knowledge and skills to do behavioural work with families [17].

### Rationale

The EPaS 2014 programme differs from other home-visiting programmes in a number of ways. Firstly, the programme specifically targets child behaviour problems. Many traditional home-visiting programmes offer a variety of services to families and do not generally target one specific aspect of family life. Secondly, the EPaS programme targets older children (3 to 4 years) than traditional home-visiting programmes that generally target children from birth to approximately 2 years. Thirdly, the programme is based on a theoretical approach to working with parents that is underpinned by social learning theory [22, 23] whereas traditional home-visiting programmes focus more as methods of service delivery as opposed to a theoretical approach [12].

### Aims and objectives

The overall aim of this trial is to conduct a multicentre, pragmatic randomised controlled trial (RCT) of the effectiveness of the EPaS 2014 programme delivered to parents of young, 3- and 4-year-old children with significant behavioural problems by health visitors by comparing it to a treatment as usual, waiting-list, control group.

The key objectives are to determine whether the EPaS 2014 programme produces statistically significant improvements in parent-reported child behaviour problems when compared to a waiting-list control group; to determine whether the EPaS 2014 programme produces any changes in secondary outcomes (observed child and parent behaviour, self-reported parental behaviour and parental depression); to determine whether child behaviour outcomes are mediated by change in parenting behaviour and/or change in parental depression; and to determine whether outcomes are moderated by risk factors such as single parents, teenage parents, poverty, and low parental education level. The study hypotheses are:

i. that the EPaS 2014 training will enable health visitors to work effectively in supporting parents of children with behaviour problems to reduce child behaviour problems.
ii. that the EPaS 2014 training will enable health visitors to bring about positive changes for parents of children with behaviour problems, including improvements in parental depression and parenting skills.

## Methods/Design

### Trial design

A pilot pragmatic, multicentre randomised controlled trial will be carried out to evaluate the effectiveness of the EPaS 2014 programme. Eligible participants will be randomly allocated to receive EPaS 2014 or to a waiting-list control group on a 1:1 ratio.

### Setting

This study will be conducted in real-world settings. Participants will be recruited from four centres in England and Wales: North West Wales (Anglesey and Gwynedd); Central North Wales (Conwy and Denbighshire); North East Wales (Flintshire and Wrexham); and Shropshire (Shrewsbury).

### Participants

Sixty health visitors across the four centres will be recruited. Each health visitor will identify 2 parents of children aged 30 to 48 months from their own caseloads whose parents are reporting their child as having significant behavioural problems identified by the child scoring at or above the clinical cut-off on the parent-reported Eyberg Child Behaviour Inventory (ECBI) [35]. Each health visitor will be required to identify two families (n = 120). Informed consent will be obtained from every participant, including health visitors and parents.

### Eligibility criteria

#### Inclusion criteria

To be eligible for the study, health visitors must have completed a Specialist Community Public Health Nursing qualification. Health visitors are deemed suitable for delivering the EPaS 2014 intervention because they have good knowledge of child development and provide regular behavioural advice to families.

Inclusion criteria for families are: (1) Parent or main caregiver of a child aged between 30 and 48 months; (2) Child scores above the clinical cut-off for behaviour problems on the ECBI (intensity scale ≥ 131 and/or problem scale ≥ 15).

#### Exclusion criteria

No exclusion criteria for health visitors.

Child exclusion criteria are: (1) any clinical diagnosis including autism and ADHD; (2) extreme learning difficulties. The exclusion criteria for parents are that they do not have a good working knowledge of Welsh and/or English.

### Recruitment

Health-visiting service managers will be approached and asked if they are interested in their staff participating in the trial. If they are, the managers are asked to identify health visitors within their service who would be interested in participating in the study. A member of the research team will then meet with interested health visitors to discuss the study and provide information regarding the commitment. Health visitors are given an Information Sheet to read and have the opportunity to ask any questions. If they agree to participate, the researcher will obtain informed consent from each health visitor. Once they have consented, they are given a pack of recruitment materials. These include the child behaviour questionnaire (the Eyberg Child Behaviour Inventory, ECBI), instructions on how to administer and score the ECBI, Note of Interest forms for eligible, interested parents to complete, freepost envelopes to send the completed ECBIs and Note of Interest forms to the research team, and a copy of the Information Sheet for parents.

Health visitors are asked to approach families on their caseloads that have a child aged between 30 and 48 months and have expressed concerns about their child’s behaviour. They ask the parents to complete the ECBI questionnaire. If they do not score above the clinical cut-off for child behaviour problems, the health visitor will thank them for their time and proceed to find an eligible family. If they score above the clinical cut-off for one of the two subscales (Intensity or Problem) the health visitor will introduce the project and ask the parent if they would be interested in taking part. If they respond positively, they are asked to complete a Note of Interest form that gives permission to the research team to contact the family to discuss the project further. The health visitor also leaves an Information Sheet for the parent to read before the visit by a researcher and then forwards the Note of Interest and completed ECBI to the research team.

On receipt of the Note of Interest, a member of the research team contacts the family to arrange a home visit to discuss the project further. The researcher ensures that the parent has read the Information Sheet and answers any questions the parent may have. If the parent is happy to continue, the researcher obtains informed consent from the parent to participate in the study. Only when the consent has been obtained will the researcher proceed to give the baseline measures to the parent.

### Intervention

The EPaS 2014 programme is based on the core components of the intensive EPaS programme [30, 31]. The programme covers assessment tools and skills, case analysis strategies and intervention components that include core parenting skills, and how to engage parents as collaborators in strategies to help address common childhood behavioural problems such as sleeping, eating, tantrums, and non-compliance.

Health visitors will complete 3 days of training, each approximately 1 month apart. The content for each training day is as follows:

1. Assessment procedures - The programme describes a standardised assessment procedure that includes a range of assessment tools including interview schedules, questionnaires, and observation tools. Health visitors will use the assessment tools to collect information about the family, their current circumstances, the specific child problem behaviours, the child’s skills and strengths, and their goals. This part of the programme takes three in-home sessions to complete.
2. Case analysis - The programme teaches how to produce a case analysis using the information collected in the assessment sessions. It involves using the information to develop an understanding of the problem, its history and current function, the assets available in the situation that will support change, and some potential short- and longer-term goals. The case analysis is shared with the family and an intervention contract is agreed. This part of the programme is undertaken in one in-home session.
3. Intervention strategies - The programme introduces intervention strategies that parents could use to achieve their short- and longer-term goals. Parents are asked to undertake assignments and keep records about their efforts to achieve weekly goals that clarify whether the intervention strategies are effective. Intervention strategies focus on teaching replacement behaviours. Example intervention strategies include praising behaviour that the parent wants to see more of, ignoring unwanted behaviours, setting limits for the child, rewards and consequences. This part of the programme can take between six to eight in-home sessions to complete, depending on the type and number of problem behaviours being targeted.

An experienced clinician (the second author) who developed the EPaS programme will conduct the training. After completing the first day of training, health visitors will begin visiting an intervention family weekly for up to 12 1-hour in-home sessions. The total number of in-home visits may vary between families depending on the complexity of the problem behaviours being targeted. Health visitors are asked to keep a record of the number of visits completed with the intervention family. All intervention resources are provided including a detailed training manual, the assessment tools for the information-gathering sessions, and packs of carbonated paper for drawing up record sheets and writing weekly targets for families. Envelopes and stamps are also given to the health visitors so they can send things to parents such as appointment letters, and so that parents can send completed records to their health visitor for feedback such as record sheets, completed assessments, and so on. Control families receive treatment as usual during this first phase and are offered the treatment 6 months later. During the 6-month wait the control group will receive usual care from the health visitors. Control families can contact their health visitor if any behavioural issues become problematic for them. This can consist of targeting problem behaviours such as sleeping, eating, and toileting using standard behavioural techniques. Control families will complete all outcome measures at the same time as the intervention group, approximately 6 months postbaseline.

### Intervention fidelity

Health visitors will be provided with a detailed training manual that they will be required to follow in their home visits. All of the assessment tools they have used during the first three in-home sessions will be reviewed during the second training day and used to formulate a case analysis and intervention goals. Intervention targets and strategies will be reviewed during the third training day.

Due to the design of the study whereby health visitors each have one intervention family and one control family, a contamination procedure will be put in place. To monitor potential contamination, health visitors will be required to keep a record of the frequency of in-home visits including whether they have seen the control family as part of usual care. If high levels of contamination are found, this information will be added to the analysis as a controlling variable. Also, researchers are blind to participant allocation; however, it is possible that participants may reveal their allocation to the researchers at the follow-up data collection visit. Researchers will be asked to record if unmasking has occurred and, again, if high levels of unmasking are found, a variable will be added to the analysis to control for this.

### Study outcomes

#### Screen

A parent-reported measure will be administered to determine the eligibility of children for inclusion in the study. The measure is the ECBI, a 36-item inventory completed by the parent for the assessment of frequency and intensity of behavioural problems in children aged 2 to 16 years [35]. Only children who score above the clinical cut-off on either the intensity subscale (≥131) or the problem subscale (≥15) will be eligible to participate. The questionnaire demonstrates good stability and homogeneity, with reliability coefficient of .86 for test-retest and .98 for internal consistency [36]. The ECBI has shown good convergent validity with scores being significantly correlated with scores on the Child Behaviour Check List [37]. Health visitors will be responsible for collecting and scoring this data for screening purposes only. Follow-up ECBI data will be collected by researchers blind to condition allocation.

### Primary outcome

The primary outcome is to establish whether there is a significant change in child behaviour from baseline to follow-up in the parent-reported ECBI.

### Secondary outcomes

The following secondary outcomes will be collected at both time points by the research team:

- Child hyperactive behaviour measured on the Conners Abbreviated Parent-Teacher Questionnaire [38]. This is a ten-item scale that comprises of the most highly loaded symptoms from the factor scales of the Conners Parent and Conners Teacher Rating Scales. Responses are rated on a 4-point scale ranging from 1 (not at all) to 4 (very much). A clinical cut-off score for hyperactivity is recommended as 15 [38].
- Observation of parent–child interaction, based on the categories from the Dyadic Parent–child Interaction Coding System (DPICS) [39]. Six parent and four child categories are employed, summarised in terms of parent positive behaviour, parent negative behaviour, child positive behaviour, and child deviance. Observational coding is continuous and records the total frequency of each behaviour per specified interval. For this study, the primary caregiver is observed interacting with their child in their own home for 30 minutes. The DPICS has shown good reliability as evidenced by a number of studies [5, 18]. Inter-rater reliability levels will be assessed during this study (20% of all observations at both time points)
- Negative parenting practices measured on the Arnold-O’Leary Parenting Scale [40]. This is a 30-item inventory that includes 3 subscales: laxness, over-reactivity, and verbosity. Responses are recorded on a seven-point scale anchored between two alternative responses to a particular situation. The questionnaire has shown adequate internal consistency (α = .63 to .84) and good test-retest reliability (*r* = .79 to .84) [40]
- Parental depression measured on the Beck Depression Inventory II (BDI) [41]. This is a 21-item inventory designed to assess the severity of characteristic symptoms and attitudes associated with depression. Each item contains 4 possible responses ranging from 0 (for example, I do not feel sad) to 3 (for example, I am so sad or unhappy that I cannot stand it). The clinical cut-off scores for this measure are as follows: minimal (0 to 13), mild (14 to 19), moderate (20 to 28), and severe (29 to63). The BDI has shown high internal consistency (α = .92), good test-retest reliability (*r* = .93), and good convergent validity (*r* = .93) [41]

An additional secondary outcome, parental satisfaction with the intervention, will be collected by the health visitors during their last in-home sessions with the parents. This is to ensure that the research team remains blind to participant group allocation.

### Demographic information

Demographic data will be collected at baseline only from all the participating health visitors and families. The questionnaires will cover the following demographic information:

- Health visitors - age, gender, number of years working as a health visitor, local area of employment, number of years working in local area, any relevant post-qualification training
- Families - age of parent and child, gender of parent and child, parent’s relationship to child, parent’s age at birth of first child, parent’s current relationship status, partner’s relation to child, housing situation, employment status, income, parent’s level of education, and whether they have attended a parenting course previously

### Mediators

Factors that have previously been shown to mediate change in similar programmes [42, 43] will be investigated. These include change in parental behaviour, as measured by the Arnold-O’Leary Parenting Scale and the parenting behaviour categories of the DPICS observation tool, and change in maternal depression, as measured by the BDI.

### Moderators

The possible moderating role of high-risk factors (using the demographic questionnaire) such as poverty, unemployment, poor housing, single parenthood, young parenthood and lack of parental education as well as the presence of maternal depression (using the BDI), and other indicators of poor outcome will be investigated.

### Data collection

Parent measures will be collected during home visits by the research team, including observation of the parent–child interaction during a free play situation. Each parent will receive a gift (a children’s book) on completion of measures at each time point. The health visitor measure (health visitor demographic questionnaire) will be collected at the first day of EPaS 2014 training.

Research staff will be trained in coding the DPICS observational tool until 80% inter-rater reliability is achieved on all categories. At least 20% of observations at each time point will be coded simultaneously by two coders to establish inter-rater reliability. Frequent practice and trouble-shooting meetings will be held to maintain high reliability levels.

### Sample size

Previous research has shown the EPaS programme to be effective for families of children with behaviour problems [33, 34]; however, these studies were limited by small sample sizes and lack of a randomised control comparison. For the current study, to detect an effect size of 0.55 standard deviation on the ECBI at 80% power and 5% significance level, a total of 55 families in each condition would be required. With a 10% drop-out rate the estimated sample size increases to 60 families in each condition. Whilst acknowledging that an effect size of 0.55 standard deviation is optimistic, due to limited funds and time a larger sample would be difficult to recruit.

### Randomisation

On completion of baseline data collection, parents will be randomised to either an intervention or a waiting-list control condition on a 1:1 ratio. The process will be done within health visitors so that each health visitor has one intervention family and one waiting-list control family. The randomisation process will be undertaken by the second author using an online randomisation programme with random permuted blocks [44].

### Blinding

Due to the nature of the study, it is not possible to have a completely blinded design. Parents will know to which condition they have been allocated. Health visitors will also be aware of which participant is in the intervention and which in the waiting-list control condition. However, the research team undertaking the data collection will be blind to participant group allocation throughout the study. Baseline measures will be collected prior to randomisation and parents and health visitors will be asked not to reveal the group allocations to the research team at follow-up. A contamination procedure will be put in place if participants reveal their allocation to the researchers.

### Statistical analyses

Baseline characteristics of the sample (health visitor, parent, and child) will be analysed and checked for differences (if any) between the two conditions, intervention and waiting-list control and any differences will be recorded.

The main analyses will be performed using the entire intention-to-treat population. The primary outcome measure, the change in the scores on the parent-reported ECBI from baseline to follow-up, will be calculated for each individual and compared between conditions using multiple linear regressions, controlling for any differences in sample characteristics at baseline and the baseline scores on the ECBI. Study site will also be controlled for in the analyses to assess any effects of clustering.

Secondary outcome measures that assess changes from baseline to follow-up in child behaviour, parental practices, and parental depression will also be analysed using multiple linear regressions, controlling for any differences in sample characteristics at baseline, baseline scores of the relevant outcome measure, and study site. In addition to the intention-to-treat analyses, per-protocol analyses will be conducted on data from participants who have remained in the study and have completed measures at all time points.

### Mediational analysis

Exploratory mediational analyses will examine the extent to which changes in child behaviour problems (as measured by the ECBI) are determined by the effects of the intervention on parent behaviour (as measured by the parenting behaviour categories of the DPICS observation tool and/or the Arnold-O’Leary Parenting Scale) and parental depression (as measured by the BDI). Analysis will be conducted using regression approaches with bootstrapping, as recommended by Dearing and Hamilton [45] using SPSS (SPSS Inc., Chicago, IL, USA) macros written by Preacher and Hayes [46].

### Moderator analysis

Indicators of poor outcome, as assessed by a demographic questionnaire, will be included in the regression models to determine whether any risk factors moderate the effect of the intervention on primary and secondary outcomes. Risk factors include lone parent, poverty, teenage parent, unemployment and low education level.

The presence of parental depression will be assessed using the BDI. This score will be collected at baseline and an interaction term (BDI * intervention group) will be included in the regression model to determine whether the intervention is less beneficial for children whose main caregiver demonstrates symptoms of depression.

### Missing data

Missing data will be managed using multiple imputation (MI). This has been found to be the most accurate method of dealing with missing data, regardless of whether it is missing at random or not [47]. Imputation strategies for MI will be reported and justified, and imputed data for MI analysed as part of a sensitivity analysis.

### Ethical approval

The study has received approval from the North Wales Research Ethics Committee (REC) and the School of Psychology, Bangor University REC (14/WA/0187).

## Discussion

This trial will provide important information on the effectiveness of an enhanced version of the EPaS programme, a one-to-one intervention to address behaviour problems in young children. The effects of the intervention on child behaviour, parenting behaviour, and parental depression will be assessed. This is a timely project when considering the rising levels of behaviour problems and the government’s focus on the importance of early intervention [1, 48].

One of the challenges of conducting this research will be the recruitment of ‘hard to reach’ families. These families can be difficult to work with due to their lack of engagement with services and/or difficulties accessing services. This is why health visitors will be identifying families for the trial as well as delivering the intervention. Other research with parenting programmes has shown that health visitors are effective in identifying parents in need of support for their child’s behaviour problems, with 81% of families identified agreeing to a visit from a researcher and 93% of those families giving informed consent [18]. Health visitors will identify families from their own caseloads so they should already have a good relationship with the families. The parents may also feel more willing to take part knowing that they will be working with their own health visitor. Health visitors will be fully aware of the details of the study and will be briefed on the best means of presenting the study in a positive way to parents.

This is the first rigorous evaluation of the EPaS 2014 programme and will potentially be a valuable addition to the child behaviour problem literature. It is hypothesised that EPaS 2014 will improve a range of outcomes, including child behaviour, parent behaviour, and parental depression, for families with a young child identified with behavioural problems. If significant results are found, the intervention may be available for use by health visitors on a more regular basis.

## Trial status

The EPaS 2014 trial is ongoing. Recruitment in the first centre commenced in August 2014. Participants from the fourth centre are expected to complete their 6-month follow-up assessment in February 2016.

## Abbreviations

ADHD: attention-deficit hyperactivity disorder
BDI: Beck Depression Inventory II
BMA: British Medical Association
CAMHS: Child and Adolescent Mental Health Services
CHPP: Child Health Promotion Programme
DPICS: Dyadic Parent–child Interaction Coding System
ECBI: Eyberg Child Behaviour Inventory
EPaS: Enhancing Parenting Skills
IT: intensive treatment
MI: multiple imputation
RCT: randomised controlled trial
REC: Research Ethics Committee
WF: Waterloo Foundation
